# Risk Factors Associated with Dentofacial Anomalies [Including Malocclusion] in Adults

**DOI:** 10.3390/bioengineering13010064

**Published:** 2026-01-07

**Authors:** Osvaldo Erik Sanchez-Hernandez, Daniel Lopez-Hernandez, Leticia Brito-Aranda, Aleli Julieta Izquierdo-Vega, Luis Beltran-Lagunes, Gabriela Patricia Fuentes-Torres, Perla Veronica Salinas-Palacios, Julio Cesar Ortega-Lopez, Maria de los Angeles Lopez-Sanchez, Edgar Estaban Torres-Garcia, Guadalupe Jacqueline Flores-Morales, Tabata Gabriela Anguiano-Velazquez

**Affiliations:** 1 Area Academica de Medicina, Instituto de Ciencias de la Salud, Universidad Autonoma del Estado de Hidalgo, Hidalgo 42090, Mexico; osvaldo_sanchez@uaeh.edu.mx (O.E.S.-H.); aleli_izquierdo11168@uaeh.edu.mx (A.J.I.-V.); 2 Clínica de Medicina Familiar “División del Norte”, Instituto de Seguridad y Servicios Sociales de los Trabajadores del Estado, Ciudad de México 04840, Mexico; 3 Centro de Investigación y de Educación Continua Sociedad Civil, Ciudad Nezahualcóyotl 57820, Mexico; letty.brito@gmail.com (L.B.-A.); dr.luisbeltran007@hotmail.com (L.B.-L.); jacky_gfm@hotmail.com (G.J.F.-M.); 4 Clínica de Especialidades Dentales “Dr. Honorato Villa Acosta”, Instituto de Seguridad y Servicios Sociales de los Trabajadores del Estado, Ciudad de México 06900, Mexico; gfuentes@issste.gob.mx; 5 Centro Médico Nacional “20 de Noviembre”, Instituto de Seguridad y Servicios Sociales de los Trabajadores del Estado, Ciudad de México 03104, Mexico; perla.salinas@issste.gob.mx; 6 Clínica de Medicina Familiar “Gustavo A. Madero”, Instituto de Seguridad y Servicios Sociales de los Trabajadores del Estado, Ciudad de México 07020, Mexico; juliocesar.ortegalopez@yahoo.com; 7 Clínica de Detección y Diagnóstico Automatizado, Instituto de Seguridad y Servicios Sociales de los Trabajadores del Estado, Ciudad de México 01030, Mexico; 8 Subdirección de Prevención y Protección a la Salud, Dirección Médica, Instituto de Seguridad y Servicios Sociales de los Trabajadores del Estado, Ciudad de México 14070, Mexico; etorres@issste.gob.mx; 9 Unidad de Medicina Familiar No. 03, Órgano de Operación Administrativa Desconcentrada Distrito Federal 2 Norte, Instituto Mexicano del Seguro Social, Ciudad de México 06600, Mexico; thathis7@hotmail.com

**Keywords:** dentofacial anomalies, neurological disorders, primary healthcare

## Abstract

**Background**: Dentofacial anomalies, including malocclusion, emerge from the interplay of genetic, clinical, and environmental determinants. Understanding the factors associated with these anomalies is crucial at the primary care level. Our study aimed to determine the possible associated factors with dentofacial anomalies in patients attended at the primary care level. **Methods**: A multivariate logistic regression model was applied to a primary care population, with the presence of dentofacial anomalies as the dependent variable. Independent variables included age and selected clinical conditions of dental and neurological origin. **Results**: Age was inversely associated with dentofacial anomalies (OR = 0.991; 95% CI 0.985–0.998; *p* = 0.013). Significant clinical factors included vertigo (OR = 2.59; 95% CI 1.42–4.71; *p* = 0.002), hearing loss (OR = 4.34; 95% CI 2.44–7.72; *p* < 0.001), trigeminal neuralgia (OR = 8.54; 95% CI 3.22–22.67; *p* < 0.001), Bell’s palsy (OR = 9.19; 95% CI 4.01–21.04; *p* < 0.001), caries limited to enamel (OR = 17.92; 95% CI 12.99–24.71; *p* < 0.001), and acute gingivitis (OR = 10.64; 95% CI 5.61–20.20; *p* < 0.001). **Conclusions**: Both oral and neurological conditions showed strong associations with dentofacial anomalies. The model identified key factors that may facilitate early detection and guide the development of targeted preventive strategies in oral health practice and policy, supporting the integration of multidisciplinary approaches to patient care.

## 1. Introduction

Dentofacial anomalies, including malocclusion, represent complex conditions that arise from the interplay of genetic, congenital, environmental, and neuromuscular factors influencing craniofacial growth and occlusal development [1,2,3,4]. Beyond malocclusion, several congenital craniofacial conditions share developmental pathways with dental anomalies. Cleft lip and palate have been associated with genetic variants in paired box 9 (PAX9) and muscle segment (MSX1), genes that are also implicated in tooth agenesis and orofacial deformities [5,6]. The well-established involvement of MSX1 in the aetiology of craniofacial malformations underscores that these conditions do not manifest as discrete or isolated entities but instead exist along a continuum of congenital and developmental disturbances affecting the craniofacial complex [7,8].

Odontogenesis is regulated by a complex network of homeobox genes and mesenchymal signalling molecules that orchestrate craniofacial morphogenesis [4]. Key genes include muscle segment (MSX1 and MSX2), which determine the spatial positioning and subsequent development of tooth buds, while distal-less (DLX1 and DLX2) and BARX1 are essential for molar formation [4]. The transcription factor PAX9 plays a pivotal role in tooth morphogenesis by enabling the inductive capacity of the dental mesenchyme and regulating the expression of genes including bone morphogenetic protein (BMP), MSX1, and the lymphoid enhancer-binding factor 1 (LEF1) [4]. Moreover, multiple signalling pathways, including tumour necrosis factor (TNF), fibroblast growth factor (FGF), BMP, sonic hedgehog (SHH), Wnt, Notch, and nuclear factor kappa B (NF-kB), interact to initiate critical roles in regulating tooth organogenesis [4,9,10,11,12]. In addition, malocclusion, both skeletal and dental, could be transmitted through inheritance, and chromosomal defects account for approximately 10% of all malocclusions [3]. Dental anomalies exhibit a multifactorial, polygenic, and epigenetic nature [4,13,14,15] that affects aesthetics and masticatory function. Alongside genetic determinants, environmental and functional factors also contribute substantially to the development of dentofacial anomalies [16,17,18,19]. Environmental factors associated with developmental defects include ingestion of chemicals such as fluorides, tetracyclines, dioxins, and thalidomide; prematurity; low birth weight; severe malnutrition; neonatal hypocalcaemia; vitamin D deficiency; bilirubinaemia; thyroid and parathyroid disturbances; maternal diabetes; neonatal asphyxia; severe infections; and metabolic disorders [14]. Beyond systemic influences such as maternal metabolic alterations, neonatal complications, or exposure to teratogenic agents [14], clinically relevant behavioural and functional habits—such as tongue thrusting, mouth breathing, prolonged oral habits, the practice of wind or string instruments (e.g., violin), and certain sports involving craniofacial impact or postural strain—play an important role in occlusal development [20,21,22,23,24,25,26,27,28,29]. Moreover, the facial asymmetry associated with Bell’s palsy may result in dysarthria and oral incompetence, which are frequently observed clinical findings in patients with dentofacial anomalies [30,31]. Those anomalies contribute to periodontal deterioration, temporomandibular dysfunction, and impaired quality of life [32], reflecting the complex interaction between genetic determinants and environmental influences. Understanding the underlying risk factors associated with these anomalies is essential in orthodontic biomechanics, where the precise control of force systems determines treatment success and the maintenance of healthy periodontal tissues [33,34,35]. Thus, a thorough understanding of the systemic, neurological, and dental risk factors associated with dentofacial anomalies has both clinical and biomechanical relevance and can contribute to refining biomechanical analyses prior to clinical application and to optimising the safety of orthodontic interventions [35,36,37,38].

At the public health level, identifying risk factors associated with malocclusion and related anomalies supports the development of preventive and early detection strategies in primary care settings. Recognising these determinants also informs the creation of evidence-based policies and investment in community oral health programmes aimed at reducing the burden of dentofacial anomalies. Therefore, this study aims to determine the risk factors associated with dentofacial anomalies, including malocclusion, in adult patients attending primary care services. By integrating epidemiological evidence with biomechanical principles, these findings may inform both orthodontic practice and oral health policy, contributing to the design of safer and more effective preventive and therapeutic strategies.

## 2. Materials and Methods

### 2.1. Study Design and Data Collection

The study was designed as a population-based, cross-sectional, and analytical investigation using a previously published secondary dataset [39]. The data included patients from Mexico who attended at the primary care level (“División del Norte” Family Medicine Clinic [FMC], ISSSTE—Instituto de Seguridad y Servicios Sociales de los Trabajadores del Estado—by its acronym in Spanish, Mexico City, Mexico) [39]. The dataset was extracted from the Medical Financial Information System (SIMEF, Mexico City, Mexico—System), which systematically records outpatient consultations conducted by healthcare personnel [39]. The dataset included medical records from January to December 2022 [39]. The complete database consists of 73,974 records corresponding to 17,918 patients of all age groups [39]. For this study, only records involving patients within 20 years old and over were included (n = 16,197). The study was conducted from 1 May to 31 August 2025.

### 2.2. Patient Selection and Study Population

The data collection and selection process was conducted through a structured and systematic procedure to ensure data accuracy, consistency, and reliability. Initially, information was extracted and downloaded monthly—from January to December—using Excel files generated by the SIMEF System. Subsequently, all monthly datasets were consolidated into a single comprehensive database, and each individual patient record was cross-checked to verify accuracy and prevent duplication.

A thorough review of the merged dataset was then performed to confirm the integrity and internal consistency of the information. Once the validation process was completed, the database was screened to identify records meeting the predefined inclusion criteria, while those not meeting these criteria were excluded.

Following this selection process, a complete census was obtained, comprising a total of 16,197 subjects aged 20 years and over. This methodological approach ensured the precision, quality, and reliability of the extracted data. Finally, the verified information was stored in a unified Excel workbook, which served as the main statistical database for subsequent analyses.

The study included patients of both sexes aged 20 years and older who had at least one medical consultation recorded in the SIMEF System during the study period. Only records containing complete and verifiable information were considered eligible. These included patient identification data—such as name, file number, sex, and beneficiary type—as well as clinical information comprising the diagnosis code based on the International Classification of Diseases, 10th Revision (ICD-10) and the corresponding consultation dates. Conversely, the exclusion criteria comprised patients of both sexes who were younger than 20 years, those whose records in the SIMEF System were incomplete, and cases identified as duplicates or containing data inconsistencies. These criteria ensured that only reliable and complete information was included in the final analysis, thereby strengthening the validity and quality of the study results.

### 2.3. Sampling and Data Procedure

A census sampling method was employed, incorporating all eligible records from the validated dataset. This approach ensured that only patients with complete and internally consistent records were included in the study, thereby guaranteeing the accuracy and reliability of the analysed information.

From the original database, which contained a total of 2491 ICD-10 diagnostic codes, a subset of 2355 codes corresponding to adults 20 years old and over was identified and analysed. In addition, four new variables were created to represent the presence of obesity, dyslipidaemia, asthma, and dentofacial anomalies [including malocclusion]. For obesity, the following ICD-10 codes were included: E66.0, E66.8, and E66.9. For dyslipidaemia, the selected codes were E78.0, E78.1, E78.2, and E78.5. For asthma, the following codes were considered: J45.0, J45.1, J45.8, J45.9, and J46.X. Finally, for dentofacial anomalies, the next codes were incorporated: K07.0, K07.2, K07.4, K07.5, K07.6, K07.8, and K07.9 (Excl.: hemifacial atrophy or hypertrophy [Q67.4] and unilateral condylar hyperplasia or hypoplasia [K10.8]).

To estimate the prevalence of the selected comorbidities, all ICD-10 diagnostic codes were recoded as dichotomous variables, assigning a value of 0 to indicate the absence of the disease and 1 to indicate its presence. Each comorbidity was analysed independently to ensure precision in the estimation process.

This methodological strategy enabled a robust estimation of disease prevalence and facilitated the characterisation of comorbidity patterns within the study population, ensuring the consistency, comparability, and analytical validity of the obtained data.

### 2.4. Variables and Statistical Analysis

All complete patient records were included to ensure a comprehensive and reliable dataset for analysis. The variables examined comprised age (in years, and we created the variable of age group: early adulthood [EA: 20–39 years old], midlife [ML: 40–59 years old], and elderly population [EP: ≥60 years old]), sex (male or female), and comorbidities, which were classified according to the ICD-10. Categorical variables were expressed as absolute frequencies and percentages, whereas quantitative variables were summarised using mean, standard deviation (SD), and interquartile range (IQR). When applicable, results were reported with their corresponding 95% confidence intervals (95% CI). For bivariate analyses, categorical variables were compared using the Pearson chi-square test (χ^2^), the likelihood ratio chi-square test, or Fisher’s exact test, depending on data distribution and expected frequencies. Quantitative variables were compared between independent groups using Student’s *t*-test or the median test, as appropriate. A two-tailed *p*-value < 0.05 was considered statistically significant. Multivariate logistic regression models were applied to identify factors associated with dentofacial anomalies. In each model, odds ratios (ORs) and their corresponding 95% confidence intervals were calculated to quantify the strength and precision of the associations. This approach allowed for adjustment of potential confounding variables and the identification of independent predictors. An OR greater than 1 indicated a higher likelihood of dentofacial anomalies, whereas an OR less than 1 denoted a lower likelihood. A *p*-value < 0.05 (two-tailed) was considered statistically significant in all analyses.

### 2.5. Ethical Considerations

The present study was conducted in compliance with the Good Clinical Practice Guidelines, national regulations, and the principles outlined in the Declaration of Helsinki for research involving human subjects. The study protocol received approval from two institutional bodies: the Research Committee and the Research Ethics Committee (approval number MFDN/SM//EZ/315/2024, dated 6 February 2024) of the FMC “División del Norte.” The Research Ethics Committee functions as the Institution Bioethics Committee.

All data were managed under strict confidentiality standards. Access to the complete dataset, including identifiable patient information (e.g., names), was restricted exclusively to the main investigators. In order to ensure anonymity, patient names were replaced with unique identification codes, allowing the linkage of records to specific individuals without disclosing their identities. This anonymisation process safeguarded participant privacy and upheld the ethical integrity of the research. The anonymisation procedure was completed prior to sharing the dataset for statistical analysis with collaborating researchers. After the analyses were performed, only aggregated and processed statistical data was made available to the rest of the research team. This protocol ensured that all stages of data handling adhered to ethical principles [39].

## 3. Results

### 3.1. Characteristics of the Study Population

A total population of 16,197 subjects was included. Most of the participants are females and elderly.

The average age of the study population was around 56 years old, with an age range of 88 years and with a similar median age (Table 1). A statistically significant difference was observed between sexes (*p* < 0.001): females had a higher average age compared with males (Table 1). Likewise, the median age among females was higher than that of males (Table 1). When stratifying the population according to dentofacial anomaly status, an overall prevalence of 2.3% was identified within the cohort (n = 374; 95% CI 2.1–2.5).

### 3.2. Demographic Profile of Patients with Dentofacial Anomalies

Among patients diagnosed with dentofacial anomalies, 238 were females and 136 were males. The mean age of patients with dentofacial anomalies was slightly lower and statistically different (*p* = 0.009) compared with individuals without the condition (Table 2). However, the median age of patients with dentofacial anomalies was similar compared with their counterparts (Table 2) without dentofacial anomalies (*p* = 0.322). This comparable age distribution between groups indicates that the study population was well balanced with respect to age, minimising potential bias related to this demographic variable.

On the other hand, the epidemiological distribution of dentofacial anomalies cases across age groups revealed a clear age-related trend in prevalence. The EA group exhibited the lowest proportion of dentofacial anomaly cases. In contrast, the EP accounted for over two-fifths of all cases (Table 3). Therefore, our findings indicate that the prevalence of dentofacial anomalies increases progressively with age, reaching its highest level among the elderly population. This pattern suggests that age-related factors—such as cumulative dental wear, bone and periodontal changes, and loss of occlusal stability—may contribute to the higher occurrence of dentofacial anomalies observed in the elderly population. However, the epidemiological distribution of dentofacial anomalies (including malocclusion), showed age- and sex-related variations within the study cohort. Although the differences were not statistically significant, distinct demographic patterns were observed between females and males. Among females, the highest proportion of dentofacial anomalies was found in the ML group, followed closely by the EP, and a smaller proportion in EA group. In contrast, among males, the distribution pattern differed, with the highest prevalence observed in the EP, followed by the ML group, and the lowest prevalence in EA group. Overall, this pattern may reflect the combined influence of biological, hormonal, and behavioural factors that vary across age and sex groups.

### 3.3. Burden of Comorbidities of Patients with Dentofacial Anomalies

In the study cohort, other disorders of teeth and supporting structures (ODTSS) were the most frequent comorbidity among patients with dentofacial anomalies, followed by hypertension and diabetes mellitus. ODTSS showed a clear predominance in females, while hypertension and diabetes were similarly distributed between sexes.

Dyslipidaemias and caries limited to enamel (CLE) occupied the next positions in frequency, both exhibiting comparable proportions in males and females. Obesity was significantly more common among females, whereas leucoplakia and other disturbances of the oral epithelium (LODOE) were slightly more prevalent in males.

Moreover, COVID-19 infection and embedded teeth were observed with similar frequency in both sexes, while acute pharyngitis appeared as the least frequent comorbidity within the top ten conditions identified.

Overall, the comorbidity pattern highlights that oral pathologies such as ODTSS, CLE, and LODE coexist notably with chronic metabolic conditions including hypertension, diabetes, dyslipidaemia, and obesity. These findings suggest that dentofacial anomalies are frequently accompanied by systemic and oral alterations, with subtle but consistent sex-related differences in specific comorbidities such as ODTSS and obesity (Table 4).

### 3.4. Age- and Sex-Specific Distribution of the Principal Comorbidities in Patients with Dentofacial Anomalies

When comparing the distribution of comorbidities between males and females, several sex-related differences appear. The epidemiological distribution of comorbidities among females with dentofacial anomalies revealed that ODTSS were present in all cases (n = 102; 100.0%, 95% CI 100.0–100.0). The most prevalent medical conditions included hypertension (n = 60; 58.8%, 95% CI 49.3–68.4), diabetes (n = 53; 52.0%, 95% CI 42.3–61.7), dyslipidaemia (n = 45; 44.1%, 95% CI 34.5–53.8), CLE (n = 44; 43.1%, 95% CI 33.5–52.7), and obesity (n = 42; 41.2%, 95% CI 31.6–50.7). Additional comorbidities included hypothyroidism (n = 33; 32.4%, 95% CI 23.3–41.4), COVID-19 (n = 29; 28.4%, 95% CI 19.7–37.2), LODOE (n = 27; 26.5%, 95% CI 17.9–35.0), and acute pharyngitis (n = 26; 25.5%, 95% CI 17.0–33.9). Among males with dentofacial anomalies, ODTSS were also present in all individuals (n = 65; 100.0%, 95% CI 100.0–100.0). furthermore, the most common comorbidities were hypertension (n = 32; 49.2%, 95% CI 37.1–61.4), dyslipidaemia (n = 25; 38.5%, 95% CI 26.6–50.3), CLE (n = 25; 38.5%, 95% CI 26.6–50.3), and diabetes (n = 24; 36.9%, 95% CI 25.2–48.7). Other relevant comorbidities were: LODOE (n = 21; 32.3%, 95% CI 20.9–43.7), benign prostatic hyperplasia (n = 21; 32.3%, 95% CI 20.9–43.7), obesity (n = 17; 26.2%, 95% CI 15.5–36.8), COVID-19 (n = 15; 23.1%, 95% CI 12.8–33.3), and embedded teeth (n = 14; 21.5%, 95% CI 11.5–31.5).

From an epidemiological perspective, the comparative analysis between sexes indicates differential morbidity patterns. Females exhibited a higher prevalence of metabolic and endocrine disorders, suggesting a clustering of cardiometabolic risk factors. In contrast, males presented greater frequency of oral and genitourinary conditions. These findings underscore the influence of sex-specific biological, behavioural, and life-course determinants on the comorbidity burden among individuals with dentofacial anomalies, highlighting the need for tailored preventive and management strategies within primary care and population health frameworks.

On the other hand, the age-specific distribution of comorbidities among patients with dentofacial anomalies revealed a gradual epidemiological transition from predominantly localised and inflammatory conditions in early adulthood to chronic non-communicable diseases in later life.

In EA, the comorbidity profile was dominated by oral developmental and inflammatory disorders. Embedded teeth were the most frequent condition, followed by ODTSS. Local inflammatory diseases such as acute gingivitis, acute pharyngitis, and CLE were also frequent, reflecting transient inflammatory responses in oral and pharyngeal tissues as well. LODOE and dyslipidaemia were identified within this age group, suggesting the early onset of metabolic and mucosal alterations. Additionally, COVID-19, obesity, and gastro-oesophageal reflux disease without oesophagitis (GORDsO) were observed, representing the coexistence of infectious, metabolic, and gastrointestinal conditions even among younger adults. During ML, the pattern shifted towards a mixed burden of oral and systemic disorders. ODTSS remained highly prevalent, alongside CLE and LODOE. However, systemic metabolic diseases such as dyslipidaemia, obesity, diabetes, and hypertension became increasingly prominent. Hypothyroidism, acute pharyngitis, and COVID-19 were also present. This coexistence of oral pathologies with metabolic and endocrine disorders reflects the onset of chronic disease clustering typically observed in midlife populations. Among the EP, the comorbidity spectrum was dominated by chronic non-communicable diseases. Hypertension, diabetes, and dyslipidaemia were the leading comorbidities, representing the core of the cardiometabolic burden. CLE and LODOE persisted, though at lower frequencies compared with younger groups. Other systemic conditions—obesity, hypothyroidism, venous insufficiency, low back pain, and periapical abscess without sinus (PAsS)—reflected the increasing multimorbidity and physiological decline associated with ageing (Table 5).

In relation to among young females, the most prevalent condition was embedded teeth (K01.0, 14; 29.2% [16.3–42.0]), followed by COVID-19 infection (U07.1, 11; 22.9% [11.0–34.8]). Acute pharyngitis (J02.9, 9; 18.8% [7.7–29.8]) ranked third in frequency. Dental caries (K02.0), gingivitis (K05.0), and disorders of teeth and supporting structures (K08) shared the same prevalence (8; 16.7% [6.1–27.2]). Oral lesions (K13.2, 7; 14.6% [4.6–24.6]) followed, while urinary tract infection (N39.0, 5; 10.4% [1.8–19.1]) was less common. Obesity, anxiety disorder (F41.9), and caries of dentine (K02.1) all had identical low prevalence (4; 8.3% [0.5–16.2]), and dyslipidaemia was the least frequent condition (3; 6.3% [−0.6–13.1]). In contrast, among younger males, embedded teeth (K01.0, 9; 31.0% [14.2–47.9]) was the most common condition, followed by ODTSS (K08, 8; 27.6% [11.3–43.9]) and COVID-19 infection (U07.1, 7; 24.1% [8.6–39.7]). GORD (K21.9, 5; 17.2% [3.5–31.0]) was moderately prevalent. Moreover, obesity, gingivitis (K05.0), and other and unspecified lesions of oral mucosa (K13.7) shared a similar prevalence (3; 10.3% [−0.7–21.4]). A group of conditions including HIV disease (B24.X), dyslipidaemia, chronic sinusitis, impacted teeth (K01.1), gingival and edentulous alveolar ridge lesions associated with trauma (K06.2), disturbances of salivary secretion (K11.7), recurrent oral aphthae (K12.1), low back pain (M54.5), and prediabetes (R73.0) all showed equal low prevalence (2; 6.9% [−2.3–16.1]). Overall, in early adulthood, females exhibited slightly higher prevalence of embedded teeth and COVID-19 infection, while males showed greater prevalence of embedded and impacted teeth. Metabolic and systemic conditions such as obesity and dyslipidaemia were less frequent in this age group compared with older populations, and several oral pathologies shared similar low prevalence, reflecting the early manifestation of oral-systemic comorbidity patterns. These findings underscore the importance of preventive oral and metabolic care in young adults to mitigate long-term health consequences.

Similarly, in the female middle-aged population, the most prevalent oral condition was ODTSS (K08, 47; 48.9% [38.9–58.9]), followed by CLE (K02.0, 22; 22.9% [14.5–31.3]). Obesity (20; 20.8% [12.7–29.0]) and dyslipidaemia (20; 20.8% [12.7–29.0]) shared the same prevalence, indicating a notable metabolic burden in this group. Diabetes (17; 17.7% [10.1–25.3]) and hypertension (I10.X, 15; 15.6% [8.4–22.9]) were also observed at considerable frequencies. Hypothyroidism (E03.9, 13; 13.5% [6.7–20.4]), COVID-19 infection (U07.1, 12; 12.5% [5.9–19.1]), and acute pharyngitis (J02.9, 11; 11.5% [5.1–17.8]), were reported at moderate prevalence. Less common conditions included LODOE (K13.2, 10; 10.4% [4.3–16.5]) and other and unspecified lesions of oral mucosa (K13.7, 10; 10.4% [4.3–16.5]), while embedded teeth (K01.0, 9; 9.4% [3.5–15.2]) were the least frequent among the observed conditions. Among middle-aged males, ODTSS (K08, 12; 28.6% [14.9–42.2]) was the most common oral condition, closely followed by dental caries (K02.0, 10; 23.8% [10.9–36.7]) and dyslipidaemia (10; 23.8% [10.9–36.7]). Oral lesions (K13.2, 8; 19.0% [7.2–30.9]) were also prevalent. Obesity (6; 14.3% [3.7–24.9]), hypertension (I10.X, 6; 14.3% [3.7–24.9]), COVID-19 infection (U07.1, 6; 14.3% [3.7–24.9]), and diabetes (6; 14.3% [3.7–24.9]) were observed at similar frequencies. Acute pharyngitis (J02.9, 4; 9.5% [0.6–18.4]) and acute periodontitis (K05.2, 4; 9.5% [0.6–18.4]) were less common.

In females of older age, ODTSS (K08, 47; 50.0% [39.9–60.1]) and hypertension (I10.X, 45; 47.9% [37.8–58.0]) were the most prevalent conditions, highlighting a high burden of oral and cardiovascular morbidity. Diabetes (35; 37.2% [27.5–47.0]) and dyslipidaemia (22; 23.4% [14.8–32.0]) were also prevalent. Hypothyroidism (E03.9, 18; 19.1% [11.2–27.1]) and obesity (18; 19.1% [11.2–27.1]) occurred with similar frequency. Dental caries (K02.0, 14; 14.9% [7.7–22.1]) was less common, followed by venous insufficiency (I87.2, 12; 12.8% [6.0–19.5]). Finally, conditions with moderate prevalence included chronic periodontitis (K05.3, 10; 10.6% [4.4–16.9]), oral lesions (K13.2, 10; 10.6% [4.4–16.9]), irritable bowel syndrome (K58.9, 10; 10.6% [4.4–16.9]), low back pain (M54.5, 10; 10.6% [4.4–16.9]), urinary tract infection (N39.0, 10; 10.6% [4.4–16.9]), depressive episode (F32.9, 9; 9.6% [3.6–15.5]), and acute periodontitis (K05.2, 9; 9.6% [3.6–15.5]). Anxiety disorder (F41.9, 8; 8.5% [2.9–14.2]) and PAsS (K04.7, 8; 8.5% [2.9–14.2]) were among the least prevalent.

However, in older males, ODTSS (K08, 47; 72.3% [61.4–83.2]) and hypertension (I10.X, 45; 69.2% [58.0–80.5]) also showed the highest prevalence. Benign prostatic hyperplasia (N40.X, 35; 53.8% [41.7–66.0]) and diabetes (22; 33.8% [22.3–45.3]) were also common. Dental caries (K02.0, 18; 27.7% [16.8–38.6]) and dyslipidaemia (18; 27.7% [16.8–38.6]) were observed with equal frequency, while oral lesions (K13.2, 14; 21.5% [11.5–31.5]) were slightly less common. Obesity (12; 18.5% [9.0–27.9]) and several conditions shared moderate prevalence: venous insufficiency (I87.2, 10; 15.4% [6.6–24.2]), PAsS (K04.7, 10; 15.4% [6.6–24.2]), other and unspecified lesions of oral mucosa (K13.7, 10; 15.4% [6.6–24.2]), hearing loss (H91.9, 10; 15.4% [6.6–24.2]), and chronic ischaemic heart disease (I25.9, 10; 15.4% [6.6–24.2]). Acute pharyngitis (J02.9, 9; 13.8% [5.4–22.2]) and low back pain (M54.5, 9; 13.8% [5.4–22.2]) were less frequent.

### 3.5. Associated Factors to Dentofacial Anomalies

The logistic regression models identified distinct associated factors for dentofacial anomalies patients.

In the overall population model, age acted as a protective factor (OR = 0.99; 95% CI: 0.98–1.00; *p* = 0.013). Significant associations were observed for vertigo (H810; OR = 2.59; 95% CI: 1.42–4.71; *p* = 0.002), hearing loss (H919; OR = 4.34; 95% CI: 2.44–7.72; *p* < 0.001), trigeminal neuralgia (G500; OR = 8.54; 95% CI: 3.22–22.67; *p* < 0.001), Bell’s palsy (G510; OR = 9.19; 95% CI: 4.01–21.04; *p* < 0.001), dental caries (K020; OR = 17.92; 95% CI: 12.99–24.71; *p* < 0.001), and acute gingivitis (K050; OR = 10.64; 95% CI: 5.61–20.20; *p* < 0.001).

The female model showed a pattern closely resembling that of the total population, with the only difference being that age did not reach statistical significance (OR = 0.99; 95% CI: 0.98–1.00; *p* = 0.079). Also, significant associations were identified for vertigo (H810; OR = 3.19; 95% CI: 1.65–6.16; *p* = 0.001), hearing loss (H919; OR = 4.18; 95% CI: 1.87–9.34; *p* < 0.001), trigeminal neuralgia (G500; OR = 10.13; 95% CI: 3.47–29.53; *p* < 0.001), Bell’s palsy (G510; OR = 11.55; 95% CI: 4.38–30.47; *p* < 0.001), dental caries (K020; OR = 17.34; 95% CI: 11.56–26.01; *p* < 0.001), and acute gingivitis (K050; OR = 14.63; 95% CI: 7.14–29.99; *p* < 0.001).

In contrast, the male model included fewer variables significantly associated with the outcome. Age again did not reach statistical significance (OR = 0.99; 95% CI: 0.98–1.00; *p* = 0.059). The conditions that remained significantly associated were hearing loss (H919; OR = 4.65; 95% CI: 2.03–10.66; *p* < 0.001), Bell’s palsy (G510; OR = 7.42; 95% CI: 1.70–32.47; *p* = 0.008), dental caries (K020; OR = 19.38; 95% CI: 11.42–32.90; *p* < 0.001), and acute gingivitis (K050; OR = 4.24; 95% CI: 1.06–17.03; *p* = 0.042).

Taken together, the models demonstrate a consistent pattern in which oral diseases (K020, K050) and otoneurologic disorders (H810, H919, G500, G510) are strongly associated with the studied outcome across the total population and among women, whereas in men the associations were restricted to fewer variables with similar directionality but smaller magnitude. These findings highlight potential sex-based differences in the strength and range of associations between oral and neurological conditions within the studied population.

## 4. Discussion

A comprehensive understanding of the epidemiological burden and comorbidity profile of dentofacial anomalies is fundamental for translating orthodontic biomechanics into clinical decision-making frameworks that account for patient-specific biological and systemic contexts. This study, conducted in a large primary-care cohort of over 16,000 adults, provides insights into population-level determinants and multimorbidity patterns associated with dentofacial anomalies, supporting the rationale for age- and sex-specific biomechanical strategies in orthodontic practice.

The overall prevalence of dentofacial anomalies in this cohort (2.3%) is different from other studies [40]. Prevalence of dental anomalies worldwide varies, probably due primarily to genetic variation between ethnicities [41]. The higher proportion of affected females (63.6%) aligns with evidence suggesting sex-related differences in craniofacial growth, hormonal regulation of bone metabolism, and healthcare-seeking behaviours [42,43,44,45,46]. The observed increase in prevalence with advancing age—peaking in older adults—reflects the cumulative biomechanical effects of occlusal wear, alveolar bone resorption, and periodontal degeneration over the lifespan [47,48,49,50]. It is well established that compared with teenage patients, adult patients generally show a slower rate of tooth movement and more pronounced alveolar bone loss during orthodontic treatment, suggesting a maladaptation of alveolar bone homeostasis under orthodontic force [47,48,49]. These findings underscore that orthodontic biomechanics should not be limited to developmental malocclusions but also considered as a rehabilitative approach to preserve function and occlusal stability in ageing populations.

Additionally, comorbidity analysis demonstrated that ODTSS was the most prevalent condition (44.6%), followed by hypertension (24.6%) and diabetes (20.6%). The frequent co-occurrence of local (ODTSS, enamel caries, leucoplakia) and systemic conditions (hypertension, diabetes, dyslipidaemia, obesity) suggests that dentofacial anomalies may reflect systemic vulnerability characterised by chronic inflammation, oxidative stress, and impaired bone remodelling [51,52,53,54]. This relationship has been particularly explored in the context of osteoporosis, where chronic inflammation and oxidative stress further exacerbate bone remodelling imbalance by promoting osteoclastic activity and impairing osteogenesis [52,53]. Oxidative stress disrupts the coupling between osteoblast and osteoclast activity, leading to metabolic bone diseases and contributing to the pathogenesis of skeletal disorders, including osteoporosis [52,53,54]. Reactive oxygen species generated under oxidative stress induce apoptosis of osteocytes and osteoblasts, inhibit mineralisation, and disturb osteogenesis, ultimately resulting in bone loss and disease progression [54]. Osteoporosis and periodontitis are both global bone-related diseases that increase with age and share pathophysiological pathways involving inflammatory and oxidative mechanisms [51,52,53,54]. Since the maxilla and mandible exhibit the highest bone turnover rates to accommodate tooth eruption and mastication, they may be particularly susceptible to systemic imbalances affecting bone metabolism [51]. Moreover, periodontitis has been associated with an increased risk of cardiovascular disease, aspiration pneumonia, and severe COVID-19 outcomes [51], further reinforcing the interconnectedness between oral and systemic health. These mechanisms have direct implications for orthodontic biomechanics, as periodontal and skeletal responses to mechanical forces are modulated by metabolic and inflammatory status [55,56,57]. Mechanical forces influence periodontal health through multiple molecular and cellular pathways, including immune regulation, extracellular matrix (ECM) metabolism, bone turnover, periodontal ligament stem cell (PDLSC) activity, and non-coding RNA (ncRNA) signalling [55]. Under mechanical loading, immune defence functions are altered, leading to changes in gene expression, signalling cascades, and protease activity that regulate ECM metabolism and tissue remodelling [55,56]. The coordinated activity of osteocytes, osteoblasts, and osteoclasts is activated to maintain bone homeostasis, while ncRNAs can modulate gene expression and thereby influence tissue metabolism and regenerative potential [55].

On the other hand, the human periodontal ligament (hPDL) is constantly exposed to mechanical stimuli that can trigger inflammatory responses in resident stem cells (hPDLSCs) [56]. Toll-like receptors (particularly TLR4) play a role in transducing mechanical stress into cytokine-mediated responses, highlighting the intersection between biomechanical and immunological signalling [56]. Intermittent compressive force (ICF) modulates cell proliferation and hPDL homeostasis, partly through the activation of the receptor activator of nuclear factor kappa-B ligand (RANKL) via interleukin-1β (IL-1β), thereby influencing bone metabolism and functional adaptation. ICF can also induce periodontal tissue remodelling through the TGF-β1 signalling pathway, promoting the expression of sclerostin (SOST) and periostin (POSTN) in hPDL cells [56].

Furthermore, shear stress, one of the major mechanical stimuli generated during mastication or tooth movement, exposes hPDL cells to interstitial fluid flow that modulates their osteogenic differentiation, proliferation, and ECM remodelling [56]. This mechanical cue also affects the immunomodulatory response of mesenchymal stem cells (MSCs) via COX-2/PGE_2_ expression and regulates the release of several growth factors and cytokines, including FGF-2, BMP-2, TGF-β, VEGF, and IL-6 [56]. Similarly, cyclic tensile force (CTF), which mimics occlusal stress during mastication, upregulates the expression of inflammatory cytokines (IL-1β, IL-6, IL-8, IL-11) as well as osteogenic markers such as RUNX2, ALP, and osteocalcin (OCN) [56]. These findings suggest that CTF induces mechanosensitive genes in hPDL cells, triggering an inflammatory response that primes the bone remodelling process essential for orthodontic tooth movement [56].

Beyond PDLSCs, mechanical stress also shapes the inflammatory response of osteoblasts and osteoclasts, enhancing the production of pro-inflammatory cytokines (TNF-α, IL-1β, IL-6, IL-8), chemokines, and hormonal mediators [56]. The magnitude and duration of the mechanical stimulus determine the cytokine profile and subsequent remodelling dynamics [56]. Importantly, periodontitis arises from an imbalanced host–microbe interaction that promotes dysbiosis and destructive inflammation, with innate and adaptive immune responses driving alveolar bone resorption and connective tissue breakdown [57]. This aligns with our epidemiological findings, in which acute gingivitis emerged as a risk factor associated with dentofacial anomalies, reinforcing the interplay between inflammatory periodontal conditions and skeletal deformities. Consequently, biomechanical planning should integrate systemic health assessments to mitigate the risks of root resorption, delayed tooth movement, and periodontal breakdown, particularly in older or metabolically compromised patients.

Distinct comorbidity patterns between sexes indicate differential biomechanical susceptibilities. Females presented higher rates of metabolic and endocrine disorders—including obesity, dyslipidaemia, and hypothyroidism—whereas males exhibited a greater prevalence of oral mucosal lesions and benign prostatic hyperplasia. These differences imply that hormonal and metabolic factors influence craniofacial morphology and tissue responsiveness to orthodontic forces [42,43,44,45,46,47,48,49,50,58,59]. Moreover, oestrogen deficiency has been associated with altered alveolar bone turnover and reduced anchorage stability [60]. On the other hand, males with higher periodontal and mucosal disease prevalence may experience amplified local inflammatory responses, complicating force system design and load distribution.

Age-specific analysis revealed a clear epidemiological transition: inflammatory and developmental oral conditions dominated early adulthood, mixed oral–systemic morbidity was prevalent in midlife, and chronic cardiometabolic diseases predominated in the elderly. Biomechanically, this mirrors the shift from adaptive to degenerative tissue behaviour. Younger adults can tolerate force systems designed for active tooth movement [61]. In contrast, older adults, with reduced bone density, compromised vascularity, and multimorbidity, are likely to require a low-intensity, controlled dental force system to prevent tissue overload [62]. Integrating epidemiological and biomechanical evidence thus supports the development of age-adapted orthodontic mechanics that respect senescent tissue constraints.

The associations among dentofacial anomalies and trigeminal neuralgia, Bell’s palsy, vertigo, and hearing loss highlight an important neurosensory dimension. These findings align with the concept of craniofacial functional units, where occlusal and temporomandibular dysfunctions influence neural and vestibular systems through biomechanical and neurophysiological interconnections [63]. The temporomandibular joint (TMJ) has been described as a “neurological window” and “lever,” integrated with brainstem centres via the sensorimotor system, including the neural circuits responsible for balance, coordination, and posture [63]. Dysfunction of the TMJ or masticatory muscles may therefore reflect not only local disturbances but also remote or systemic neurological imbalances [63].

Conversely, repeated, or tonic, sensory stimulation involving the TMJ—such as that induced by occlusal splints, cranial manipulation, or myofascial therapy—can influence neuronal plasticity and may serve as a potential therapeutic approach in neuromuscular and movement disorders [63,64]. This neurofunctional coupling suggests that occlusal therapies and orthodontic interventions should be approached within a holistic biomechanical framework that encompasses neuromuscular balance, postural stability, and sensorimotor integration [63]. Furthermore, the TMJ’s bidirectional communication with the central nervous system implies that mechanical or occlusal alterations can modulate proprioceptive feedback loops, muscle tone, and even cortical excitability [63]. From a physiological standpoint, such neuro-occlusal interactions reinforce the role of the stomatognathic system as a critical interface between the musculoskeletal and neural domains, providing both diagnostic and therapeutic insights for orthodontic and temporomandibular management [63,64,65]. Additionally, malocclusion (recognised by the World Health Organisation as one of the most significant oral health problems after dental caries and periodontal disease) represents not only a structural but also a functional disturbance within the stomatognathic system [66,67]. Its global prevalence is highly variable—ranging from 39% to 93% in children and adolescents [66]. The highest rates have been reported in Africa (81%) and Europe (72%), followed by the Americas (53%) and Asia (48%) [68]. The early onset and high worldwide burden of malocclusion highlight the importance of integrating preventive and corrective strategies from childhood. Although our data encompass dentofacial anomalies, the prevalence observed in our study population was significantly lower than that reported in the international literature [66,67,68], which may reflect differences in population characteristics, diagnostic criteria, or environmental and behavioural determinants influencing craniofacial development. This evidence supports a holistic biomechanical framework encompassing neuromuscular balance, postural stability, and sensorimotor integration in orthodontic treatment planning.

On the other hand, several studies have shown that patients with temporomandibular disorders (TMD) often present with higher pain severity, which in turn affects their overall life satisfaction and sleep quality [69,70,71,72]. These effects not only compromise overall well-being but may also affect work performance and the ability to carry out daily activities [73], reinforcing the need for a comprehensive approach to pain and its consequences in this patient group. The coexistence of TMD and malocclusion has been associated with increased functional impairment, parafunctional habits (such as bruxism [mainly sleep bruxism], resting the chin on the hand, sleeping in a jaw-compressing position, jaw tension, and daytime clenching), and sleep disturbances [69,70,71,72,74,75,76], suggesting that occlusal discrepancies may contribute indirectly to pain perception and reduced well-being. Likewise, adverse oral habits such as prolonged pacifier use, thumb sucking, mouth breathing, nail biting, and bottle feeding, as well as childhood caries, significantly contribute to the development of malocclusion [77]. Although the present study did not directly assess pain or sleep parameters, the identified demographic and clinical risk factors support the relevance of biomechanical and functional pathways previously described in TMD research.

The relationship between TMD and obstructive sleep apnoea (OSA) remains highly debated [78,79]. Some reports propose that craniofacial morphology, upper airway characteristics, and specific skeletal patterns of malocclusion may influence the development of OSA [80,81,82,83]. Patients with OSA may display a narrow maxillary arch, underdevelopment and clockwise rotation of the mandible, increased anterior facial height, an inferiorly positioned hyoid bone, an enlarged soft palate, and a reduced pharyngeal airway space [81,82]. Moreover, Class III males have a higher risk of OSA or other respiratory disorders in comparison with Class III females [83]. Therefore, it is reasonable to consider that the association between dentofacial anomalies and malocclusion with OSA depends on the morphogenetic characteristics of the population under study. Our results should therefore be interpreted within this context, acknowledging that dentofacial anomalies may coexist with conditions such as TMD and OSA without necessarily establishing a causal link. In addition, it is important to note that patients with congenital conditions—including Down syndrome—frequently present with distinct craniofacial traits and a higher prevalence of Class 3 malocclusion and long lower anterior facial heights, which are factors associated with the development of OSA [84,85,86].

### Limitations and Clinical Implications

This study has several limitations that should be considered when interpreting the results. First, the cross-sectional and secondary-data design precludes any inference of causality; observed associations reflect correlation at a single point in time and cannot determine temporal sequence. Second, the assessment of dentofacial anomalies relied on routine clinical records, which may introduce variability in diagnostic criteria, misclassification, or under-reporting bias of diagnoses, since coding practices can vary between clinicians. Third, residual confounding is possible: although multivariable models adjusted for measured covariates, unmeasured factors (for example, detailed socioeconomic indicators, lifestyle variables, or severity measures) could influence the associations observed. Fourth, although the dataset represents patients attending a single primary-care clinic in Mexico City, the findings may be transferrable to populations with similar demographic and healthcare characteristics, particularly in countries with emerging economies or low- and middle-income settings that share comparable epidemiological and organisational contexts. Finally, some ICD-10 codes group heterogeneous conditions (reducing clinical granularity), and the creation of composite variables may mask subtype-specific effects. These limitations underscore the need for prospective, clinically characterised studies to validate and expand upon the present findings.

Despite these limitations, the study provides meaningful epidemiological evidence that may support clinical and public-health decision-making. The identification of demographic and systemic risk factors associated with dentofacial anomalies can strengthen early detection strategies in primary care, inform referral pathways, and guide preventive interventions aimed at reducing the burden of malocclusion and related conditions. In addition, the results highlight patient groups that may benefit from closer monitoring, multidisciplinary assessment, and targeted health education. Therefore, future studies incorporating longitudinal designs and comprehensive functional assessments are warranted to confirm these associations and enhance their clinical applicability.

## 5. Conclusions

Clinically, integrating biomechanical analysis with epidemiological risk stratification can enhance the precision of orthodontic interventions. Identifying high-risk phenotypes—such as elderly females with cardiometabolic multimorbidity or males with oral–mucosal pathology—can inform the selection of anchorage systems, force magnitude, and treatment sequencing. At the population level, these findings advocate for preventive and interdisciplinary programmes addressing both oral and systemic determinants of dentofacial anomalies within primary care frameworks.

Therefore, our findings are online that dentofacial anomalies are multifactorial clinical conditions affected by demographic ageing, sex-specific biology, and comorbidity. Future research integrating bone turnover biomarkers and longitudinal epidemiological data could further elucidate the dynamic interplay among local and systemic factors with orthodontic biomechanics.

The present findings reveal a robust and biologically plausible association between oral and otoneurologic conditions, suggesting an integrated pathophysiological link between the stomatognathic, auditory, and cranial nerve systems. Dental caries and acute gingivitis emerged as the strongest predictors across all models, while vertigo, hearing loss, trigeminal neuralgia, and Bell’s palsy demonstrated consistent associations—particularly among women. Although age showed a mild protective trend, its effect was not significant in sex-stratified analyses.

Overall, the results underscore the importance of recognising oral health disorders as potential indicators or contributors to broader neurosensory dysfunctions. The observed sex-related differences in the magnitude and scope of these associations warrant further investigation into hormonal, behavioural, and structural determinants that may mediate these interactions. From a public health perspective, these data support the integration of interdisciplinary screening and management strategies linking dental, neurological, and otorhinolaryngological care to promote early detection and holistic treatment approaches.

## Figures and Tables

**Table 1:** General Characteristics of the Study Population.

Variables	Total Population N; % (95% CI)	Females n; % (95% CI)	Males n; % (95% CI)
n (%)	16,197	10,425 (64.4%)	5772 (35.6%)
95% CI	Reference	63.6–65.1	34.9–36.4
Age, average (SD)	56.83 (16.23)	57.9 (16.23)	56.2 (16.15)
Age, median (IQR)	57 (45–69)	60 (46–70)	57 (45–68)
Age, min	20	20	20
Age, max	108	108	104
Early adulthood	2725 (16.8)	917 (15.9)	1808 (17.3)
95% CI	16.3–17.4	15.0–16.9	16.6–18.1
Midlife	6086 (37.6)	1965 (34.0)	4121 (39.5)
95% CI	36.8–38.3	32.7–35.3	38.6–40.5
Elderly population	7386 (45.6)	2890 (50.1)	4496 (43.1)
95% CI	44.8–46.4	48.8–51.4	42.2–44.2

Source: Prepared by the authors using the results from the SIMEF System database, January–December 2022. 95% CI: 95% confidence interval. Early adulthood (20–39 years old). Midlife (40–59 years old). Elderly population (60 years old and over). Independent samples *t*-test showed a significant difference in age between females and males (t = 6.748, *p* < 0.001), with a mean difference of 1.79 years (95% CI: 1.27 to 2.32). A comparison of median age between females and males was conducted using the independent-samples median test. The difference between groups was statistically significant (*p* < 0.001). Comparisons between age group and sex were performed using the likelihood ratio chi-square, which showed a statistically significant association (Likelihood Ratio χ^2^ = 73.668, degree freedom = 2, *p* < 0.001).

**Table 2:** Comparison of the demographic profile of patients with and without dentofacial anomalies.

Variables	Total Population N; % (95% CI)	Females n; % (95% CI)	Males n; % (95% CI)
Population with dentofacial anomalies			
n (%)	374	238 (63.6%)	136 (36.4%)
95% CI	Reference	58.8–68.4	31.6–41.2
Age, average (SD)	54.66 (15.82)	54.1 (15.13)	55.6 (16.99)
Age, median (IQR)	56 (43–65.25)	55.5 (44.75–65)	58 (42–69)
Age, min	20	21	20
Age, max	98	95	98
Population without dentofacial anomalies			
n (%)	15,823	10,187 (64.4%)	5636 (35.6%)
95% CI	Reference	63.6–65.2	34.8–36.4
Age, average (SD)	56.89 (16.23)	56.2 (16.17)	58.1 (16.28)
Age, median (IQR)	58 (45–69)	57 (45–68)	60 (46–70)
Age, min	20	20	20
Age, max	108	108	104

Source: Prepared by the authors using the results from the SIMEF System database, January–December 2022. 95% CI: 95% confidence interval. A comparison of age distributions between females and males with dentofacial anomalies was conducted using the independent-samples Mann–Whitney U test. The analysis indicated no statistically significant difference in age distribution between sexes (*p* = 0.353). Independent samples *t*-test showed a significant difference in age between females and males (t = 6.705, *p* < 0.001), with a mean difference of 1.80 years (95% CI: 1.28 to 2.33) within subjects without dentofacial anomalies. A comparison of median age between females and males was conducted using the independent-samples median test. In patients with dentofacial anomalies showed a non-statistically significant difference (*p* = 0.363). In subjects without dentofacial anomalies showed a statistically significant difference (*p* < 0.001). Comparisons between dentofacial anomalies and sex were performed using the Yates’ continuity-corrected chi-square, which showed a non-statistically significant association (χ^2_Y_^ = 0.059, degree freedom = 1, *p* = 0.808).

**Table 3:** Age and sex distribution of the study population with dentofacial anomalies.

Age Group	Total Population N; % (95% CI)	Females n; % (95% CI)	Males n; % (95% CI)
EA	77; 20.6% (16.6–24.6)	48; 20.2% (15.5–25.2)	29; 21.3% (14.7–27.9)
ML	138; 36.9% (32.1–42.0)	96; 40.3% (34.5–46.2)	42; 30.9% (23.5–38.2)
EP	159; 42.5% (38.0–47.3)	94; 39.5% (33.2–45.0)	65; 47.8% (39.7–56.6)

Source: Prepared by the authors using the results from the SIMEF System database, January–December 2022. 95% CI: 95% confidence interval. EA: early adulthood (20–39 years old). ML: midlife (40–59 years old). EP: elderly population (60 years old and over). Comparisons between age group and sex were performed using the likelihood ratio chi-square, which showed a non-statistically significant association (Likelihood Ratio χ^2^ = 3.588, degree freedom = 2, *p* = 0.166).

**Table 4:** Epidemiological Profile of the Ten Most Prevalent Comorbidities in Patients with Dentofacial Anomalies.

No.	Comorbidity	Total Population n; % (95% CI)	Females n; % (95% CI)	Males n; % (95% CI)
1	ODTSS *	167; 44.6%, (39.6–49.7)	102; 27.3%, (22.8–31.8)	65; 17.4%, (13.5–21.2)
2	Hypertension	92; 24.6%, (20.3–28.9)	60; 25.2%, (19.7–30.7)	32; 23.5%, (16.9–30.9)
3	Diabetes	77; 20.6%, (16.6–24.6)	53; 22.3%, (17.6–27.7)	24; 17.6%, (11.8–24.3)
4	Dyslipidaemias:	70; 18.7%, (14.7–22.2)	45; 18.9%, (14.7–24.4)	25; 18.4%, (11.8–25.0)
5	CLE	69; 18.4%, (14.4–22.5)	44; 18.5%, (13.9–23.5)	25; 18.4%, (12.5–25.0)
6	Obesity *	59; 15.8%, (12.0–19.5)	42; 17.6%, (13.0–22.7)	17; 12.5%, (7.4–18.4)
7	LODOE	48; 12.8%, (9.6–16.3)	27; 11.3%, (7.6–16.0)	21; 15.4%, (9.6–22.1)
8	COVID-19	44; 11.8%, (8.6–15.0)	29; 12.2%, (8.4–16.8)	15; 11.0%, (5.9–16.9)
9	Embedded teeth	40; 10.7%, (7.5–13.6)	26; 10.9%, (7.1–15.5)	14; 10.3%, (5.1–15.4)
10	Acute pharyngitis	37; 9.9%, (7.0–12.8)	26; 10.9%, (7.1–15.1)	11; 8.1%, (3.7–13.2)

Source: Prepared by the authors using the results from the SIMEF System database, January–December 2022. 95% CI: 95% confidence interval. No.: number. ODTSS: other disorders of teeth and supporting structures. CLE: caries limited to enamel. LODOE: leucoplakia and other disturbances of oral epithelium. COVID-19: COVID-19, virus identified. * *p* values statistically significative between sex.

**Table 5:** Age-specific epidemiological Profile of the Ten Most Prevalent Comorbidities in Patients with Dentofacial Anomalies.

No.	Early Adulthood Comorbidity n; % (95% CI)	Middle Life Comorbidity n; % (95% CI)	Elderly Population Comorbidity n; % (95% CI)
1	Embedded teeth 23; 29.9% (19.6–40.1)	ODTSS 59; 42.8% (34.5–51)	Hypertension 70; 44% (36.3–51.7)
2	COVID-19 18; 23.4% (13.9–32.8)	Caries limited to enamel 32; 23.2% (16.1–30.2)	Diabetes 53; 33.3% (26–40.7)
3	ODTSS 16; 20.8% (11.7–29.8)	Dyslipidaemia 30; 21.7% (14.9–28.6)	Dyslipidaemia 35; 22% (15.6–28.5)
4	Acute gingivitis 11; 14.3% (6.5–22.1)	Obesity 26; 18.8% (12.3–25.4)	Caries limited to enamel 28; 17.6% (11.7–23.5)
5	Acute pharyngitis 10; 13% (5.5–20.5)	Diabetes 23; 16.7% (10.4–22.9)	Obesity 26; 16.4% (10.6–22.1)
6	Caries limited to enamel 9; 11.7% (4.5–18.9)	Hypertension 21; 15.2% (9.2–21.2)	LODOE 22; 13.8% (8.5–19.2)
7	LODOE 8; 10.4% (3.6–17.2)	LODOE 18; 13% (7.4–18.7)	Hypothyroidism 19; 11.9% (6.9–17)
8	Obesity 7; 9.1% (2.7–15.5)	COVID-19 18; 13% (7.4–18.7)	Venous insufficiency 19; 11.9% (6.9–17)
9	GORDsO 7; 9.1% (2.7–15.5)	Acute pharyngitis 15; 10.9% (5.7–16.1)	Low back pain 16; 10.1% (5.4–14.7)
10	Dyslipidaemia 5; 6.5% (1–12)	Hypothyroidism 14; 10.1% (5.1–15.2)	PAsS 15; 9.4% (4.9–14)

Source: Prepared by the authors using the results from the SIMEF System database, January–December 2022. 95% CI: 95% confidence interval. No.: number. GORDsO: gastro-oesophageal reflux disease without oesophagitis. ODTSS: other disorders of teeth and supporting structures. PAsS: periapical abscess without sinus. LODOE: leucoplakia and other disturbances of oral epithelium. COVID-19: COVID-19, virus identified.

## Data Availability

The data presented in this study is not publicly available due to privacy and ethical restrictions. The dataset was obtained from the SIMEF and contains sensitive personal health information protected under data confidentiality regulations. Access to the data is therefore restricted to the principal investigators and authorised personnel only. Aggregated statistical data supporting the findings of this study are available from the corresponding author upon reasonable request and subject to institutional approval.

## Abbreviations

The following abbreviations are used in this manuscript:

ALP	Alkaline Phosphatase
BARX1	BarH-like Homeobox 1
BMP	Bone Morphogenetic Protein
BMP-2	Bone Morphogenetic Protein 2
χ^2^	Chi-Square Test
CI	Confidence Interval (95% CI)
CLE	Caries Limited to Enamel
COX-2/PGE_2_	Cyclooxygenase-2/Prostaglandin E_2_
CTF	Cyclic Tensile Force
DLX1, DLX2	Distal-less Homeobox 1 and 2
EA	Early Adulthood (20–39 years old)
ECM	Extracellular Matrix
EP	Elderly Population (≥60 years old)
FGF	Fibroblast Growth Factor
FGF-2	Fibroblast Growth Factor 2
FMC	Family Medicine Clinic
GORDsO	Gastro-Oesophageal Reflux Disease without Oesophagitis
hPDL	Human Periodontal Ligament
ICD-10	International Classification of Diseases, 10th Revision
ICF	Intermittent Compressive Force
IL	Interleukin
IL-1β	Interleukin-1 Beta
IL-6, IL-8, IL-11	Interleukins 6, 8, and 11
IQR	Interquartile Range
ISSSTE	Instituto de Seguridad y Servicios Sociales de los Trabajadores del Estado
LEF1	Lymphoid Enhancer-Binding Factor 1
LODOE	Other Disturbances of the Oral Epithelium
ML	Midlife (40–59 years old)
MSCs	Mesenchymal Stem Cells
MSX1, MSX2	Muscle Segment Homeobox 1 and 2
ncRNA	Non-Coding RNA
NF-κB	Nuclear Factor Kappa B
OCN	Osteocalcin
ODTSS	Other Disorders of Teeth and Supporting Structures
OR	Odds Ratio
PAX9	Paired Box 9 Transcription Factor
PAsS	Periapical Abscess without Sinus
PDLSC	Periodontal Ligament Stem Cell
POSTN	Periostin
RANKL	Receptor Activator of Nuclear Factor Kappa-B Ligand
RUNX2	Runt-Related Transcription Factor 2
SD	Standard Deviation
SHH	Sonic Hedgehog
SIMEF	Medical Financial Information System
SOST	Sclerostin
TGF-β	Transforming Growth Factor Beta
TGF-β1	Transforming Growth Factor Beta 1
TMJ	Temporomandibular Joint
TLR	Toll-Like Receptor
TNF	Tumour Necrosis Factor
VEGF	Vascular Endothelial Growth Factor
Wnt	Wingless-Related Integration Site Signalling Pathway
